# Subinhibitory Concentrations of Biogenic Silver Nanoparticles Affect Motility and Biofilm Formation in *Pseudomonas aeruginosa*

**DOI:** 10.3389/fcimb.2021.656984

**Published:** 2021-04-01

**Authors:** Erika Kushikawa Saeki, Amanda Yaeko Yamada, Larissa Amianti de Araujo, Laís Anversa, Doroti de Oliveira Garcia, Renan Luiz Barros de Souza, Heloísa Moreira Martins, Renata Katsuko Takayama Kobayashi, Gerson Nakazato

**Affiliations:** ^1^ Regional Laboratory of Presidente Prudente, Adolfo Lutz Institute, Presidente Prudente, Brazil; ^2^ Regional Laboratory of Bauru, Adolfo Lutz Institute, Bauru, Brazil; ^3^ Regional Laboratory of Marília, Adolfo Lutz Institute, Marília, Brazil; ^4^ University of Western São Paulo, UNOESTE, Presidente Prudente, Brazil; ^5^ Laboratory of Basic and Applied Bacteriology, Department of Microbiology, Center of Biological Sciences, State University of Londrina, Londrina, Brazil

**Keywords:** antibiofilm, antivirulence, virulence factor, metal nanoparticle, quorum sensing

## Abstract

Biogenic silver nanoparticles (bio-AgNPs) are increasingly recognized as an antibiofilm and antivirulence strategy against *P. aeruginosa*, a bacterium that causes chronic infections in immunocompromised and cystic fibrosis patients. This study aimed to investigate the effects of subinhibitory concentrations of bio-AgNPs on motility and biofilm formation in *P. aeruginosa*. Bio-AgNPs were synthesized *via* reduction of ionic silver catalyzed by cell-free culture filtrate from *Fusarium oxysporum*. A total of 17 *P. aeruginosa* isolates and strains were evaluated for swarming, swimming, and twitching motility in the presence and absence (control) of bio-AgNPs, including 10 clinical isolates from patients with and without cystic fibrosis, 5 environmental isolates obtained from the public water supply system, and 2 reference strains (PAO1 and PA14). Isolates were identified by biochemical and molecular methods. Minimum inhibitory concentrations (MICs) were determined by the broth microdilution method. Swarming, swimming, and twitching motility assays were performed in Petri dishes. Biofilm formation capacity was assessed quantitatively by the crystal violet method. MIC values ranged from 15.62 to 62.50 µM. The results showed that subinhibitory concentrations of bio-AgNPs (½ MIC, 7.81–31.25 µM) significantly increased (p < 0.05) swarming, swimming, and twitching motility in 40.0, 40.0, and 46.7% of isolates, respectively. Subinhibitory bio-AgNP treatment enhanced (p < 0.05) biofilm formation capacity in PA14 and a cystic fibrosis isolate (P11). It is concluded that subinhibitory concentrations of bio-AgNPs increased biofilm formation and swarming, swimming, and twitching motility in PA14 and some *P. aeruginosa* isolates. These virulence factors are directly involved with quorum-sensing systems. Further research should investigate the effects of AgNPs on *P. aeruginosa* quorum sensing to help elucidate their mechanism of action at subinhibitory concentrations.

## Introduction

Silver nanoparticles (AgNPs) are one of the most widely studied nanomaterials (Guo et al., 2019; Kawish et al., 2020; Saeed et al., 2020) because of their chemical stability, malleability, flexibility, high electrical and thermal conductivity, catalytic activity, relatively low cost, and, mainly, potent antimicrobial action (Durán et al., 2019). These unique properties have led AgNPs to find application in the medical industry (wound dressing, surgical instrument, and bone prosthesis production), textile industry (garment production), food industry (food packaging, countertop, and cutting board materials), and wastewater treatment (McNamara and Tofail, 2017; Gadkari et al., 2018).

AgNPs were shown to have antimicrobial properties against several pathogenic microorganisms (Scandorieiro et al., 2016; Figueiredo et al., 2019), including *Pseudomonas aeruginosa* (Liao et al., 2019; D’Lima et al., 2020). This bacterium can be found in various environments, such as hospitals, natural resources, and water supply systems (Dobias and Bernier-Latmani, 2013; Anversa et al., 2019; Esmaeili et al., 2019). *P. aeruginosa* may express different virulence factors and is closely related to healthcare-related infections and diseases in immunocompromised, cystic fibrosis, and severely burned patients. Because of these factors and its multi-antimicrobial resistance, *P. aeruginosa* constitutes a public health problem (Matar, 2018; Ahmed et al., 2019).

Chronic *P. aeruginosa* infection is established mainly through biofilm formation. Motility plays an essential role in the initial adhesion of bacterial cells to biotic and abiotic surfaces (Khan et al., 2020). Biofilm formation contributes to the survival of *P. aeruginosa* under adverse environmental conditions, provides protection against the host’s immune system, and confers antimicrobial resistance. This specific bacterial response is triggered by proximity to surfaces and involves complex chemical signaling networks, including quorum sensing (QS) (Khan et al., 2019; Olivares et al., 2020).

Biofilm development can be divided into four steps: (1) reversible adhesion of bacterial cells to a surface; (2) sessile growth and increased production, release, and detection of autoinducer molecules by QS systems; (3) biofilm maturation with the formation of mushroom-like structures enveloped by exopolysaccharides; and (4) disruption through the release of cells (Saxena et al., 2019). These important steps in *P. aeruginosa* biofilm formation depend on appendages such as flagella and type IV pili, both of which are necessary for bacterial motility (Otton et al., 2017).

*P. aeruginosa* is capable of three types of movement: swarming, swimming, and twitching. Swarming is performed on semi-solid surfaces (0.5 to 0.7% agar) *via* multicellular motion dependent on rotating helical flagella and typically involves type IV pili and a class of surfactants known as rhamnolipids (Köhler et al., 2000; Kearns, 2010). Swimming is another type of motility whereby bacteria swim on a low-viscous (0.3% agar) liquid surface using rotating flagella (Kearns, 2010; Sun et al., 2018). Twitching motility is mediated by type IV pili, occurring on solid surfaces or media with moderate viscosity (1% agar) by extension and retraction of pili (Kearns, 2010; Otton et al., 2017).

Due to the increase in multidrug resistance to antimicrobials, several studies have been carried out searching for new alternatives to interfere in the regulation of bacterial virulence and control the spread of multidrug-resistant bacteria (Shah et al., 2019; Ahmed et al., 2020; Piewngam et al., 2020). A major concern is that in these studies, subinhibitory concentrations of the compounds are used and we do not know for sure the bacterial response to these nanometals. The present study aimed to assess the effect of subinhibitory concentrations of biogenic AgNPs (bio-AgNPs) on the motility and biofilm formation capacity of *P. aeruginosa*.

## Materials and Methods

### Bacterial Isolates and *P. aeruginosa* Identification

This study was conducted at the Laboratory of Food Bacteriology and Microbiology of Adolfo Lutz Institute, Presidente Prudente, São Paulo, Brazil. A total of 15 isolates of *P. aeruginosa* from different origins were analyzed. Environmental isolates (E, P1–P5, *n* = 5) were obtained from the public water supply system of São Paulo State, Brazil. Clinical isolates from patients without cystic fibrosis (non-FC, P6–P10, *n* = 5) were obtained from the Presidente Prudente Regional Hospital, São Paulo State, Brazil. Cystic fibrosis isolates (FC, P11–P15, *n* = 5) were obtained from the University of São Paulo Clinics Hospital, Brazil. Two *P. aeruginosa* reference strains were used, PAO1 and UCBPP-PA14 (hereafter referred to as PA14). Information on bacterial isolates is summarized in **Table 1**.

**Table 1:** *Pseudomonas aeruginosa* strains and bacterial isolates (Environmental, non-CF, and CF isolates), Minimum inhibitory concentration, and 1/2 MIC of bio-AgNP used in this study.

Reference strains or isolates	Type	Origin	bio-AgNP (µM)	
			MIC	1/2 MIC
**PAO1**	C	Wound patient	62.50	31.25
**PA14**	C	Burn patient	62.50	31.25
**P1**	E	Water from the public supply system	15.62	7.81
**P2**	E	Water from the public supply system	31.25	15.62
**P3**	E	Water from the public supply system	15.62	7.81
**P4**	E	Water from the public supply system	31.25	15.62
**P5**	E	Water from the public supply system	31.25	15.62
**P6**	non-CF	Tracheal secretion	31.25	15.62
**P7**	non-CF	Pleural fluid	31.25	15.62
**P8**	non-CF	Knee wound	31.25	15.62
**P9**	non-CF	Tracheal secretion	31.25	15.62
**P10**	non-CF	Sacral area	31.25	15.62
**P11**	CF	Sputum and oropharyngeal	15.62	7.81
**P12**	CF	Sputum and oropharyngeal	15.62	7.81
**P13**	CF	Sputum and oropharyngeal	15.62	7.81
**P14**	CF	Sputum and oropharyngeal	15.62	7.81
**P15**	CF	Sputum and oropharyngeal	15.62	7.81

C, clinical isolates.

E, environmental isolates.

Non-CF, clinical isolates obtained from non-cystic fibrosis patients.

CF, clinical isolates obtained from cystic fibrosis patients.

MIC, Minimum inhibitory concentration.

Isolates were seeded on Cetrimide agar (Acumedia, USA) and M-PA-C Agar (BBL, USA) and incubated at 37 and 42°C, respectively. Identification was achieved by analysis of colony morphology and biochemical characteristics (catalase, oxidase, and glucose fermentation tests). Stock cultures were maintained at −80°C in Luria–Bertani (LB) broth (Neogen, USA) containing 20% (v/v) glycerol (Synth, Brazil).

### Molecular Confirmation of *P. aeruginosa* Isolates

All isolates biochemically identified as *P. aeruginosa* were confirmed by polymerase chain reaction (PCR) using a set of primers (forward, 5′-AATTCGGCAAATTTGCTGCG-3′; reverse, 5′-GGAGCTGTCGTACTCGAAGT-3′) specific to the *oprL* gene (209 bp) (Wong et al., 2014).

Bacterial DNA was extracted by the thermal lysis method. PCR was performed in a final volume of 20 µL containing 6.4 µL of ultrapure water, 0.8 µL of each primer (10 µM), 10 µL of GoTaq^®^ Colorless Master Mix (Promega), and 2 µL of DNA. Amplification consisted of initial denaturation at 95°C for 10 min, followed by 30 cycles of 95°C for 1 min, 52°C for 30 s, and 72°C for 1 min, and final extension at 72°C for 5 min on a Veriti thermal cycler (Applied Biosystems, IL, USA). The amplified products were subjected to electrophoresis in agarose gel (1.5%) stained with 0.1 µL/mL SYBR Safe (Invitrogen, USA) for 40 min at 100 V. Gels were visualized under ultraviolet light (296 nm) using a transilluminator (Syngene^®^, DigiGenius, MD, USA), and images were captured and processed using EOS Utility^®^ software (Canon, USA).

### Preparation of Bio-AgNPs

Bio-AgNPs were synthesized according to a method previously described by Durán et al. (2005; 2006) (Patent, 2006, PI 0605681-4A2; http://www.inpi.gov.br). Bio-AgNPs were prepared by reduction of silver nitrate catalyzed by a cell-free enzyme preparation from *Fusarium oxysporum* (strain 551). The fungal inoculum was obtained from the culture collection of the Laboratory of Molecular Genetics, Luiz de Queiroz College of Agriculture (ESALQ), University of São Paulo, Piracicaba, São Paulo State, Brazil. First, *F. oxysporum* was grown for 7 days at 28°C on medium containing 0.5% (w/v) yeast extract (Neogen, USA), 2% (w/v) malt extract (Neogen, USA), 2% (w/v) agar (Acumedia, USA), and distilled water. Then, the fungal biomass (0.1 g/mL) was mixed with sterile distilled water and incubated at 28°C for 72 h under constant stirring (150 rpm). After filtration, the cell-free filtrate was mixed with 0.01 M silver nitrate (AgNO_3_, Nuclear, Brazil) and incubated for 15 days at 28°C in the dark. Bio-AgNPs were washed with distilled water, centrifuged at 27 000 × *g* and 4°C for 30 min, and incubated in an ultrasonic bath for 30 min. Washing steps were repeated three times. Quantification of Ag was performed on an EDX-7000 energy dispersive X-ray fluorescence spectrometer (Shimadzu, Japan). Particle diameter and zeta potential were determined by photon correlation spectroscopy using a Zetasizer Nano ZS (Malvern Panalytical, United Kingdom). Transmission electron microscopy was performed at the State University of Maringá, Paraná, Brazil.

### Minimum Inhibitory Concentration (MIC) Determination

The MIC of bio-AgNPs for *P. aeruginosa* isolates was determined by the broth microdilution method in 96-well plates, as recommended by the Clinical and Laboratory Standards Institute (CLSI, 2015). Bio-AgNPs were diluted in Mueller–Hinton broth (Difco, USA) to concentrations of 1000 to 7.81 µM. After incubation at 37°C for 24 h, MIC values were defined as the lowest concentration of bio-AgNPs capable of preventing visible microbial growth. Positive (*P. aeruginosa* incubated in the absence of bio-AgNPs) and negative (bio-AgNPs and broth only) controls were used.

### Virulence Factor Analysis

#### Swarming Motility

*P. aeruginosa* isolates were grown in LB broth (Neogen, USA) for 24 h at 30°C. Then, swarming agar plates containing 1% glucose (Synth, Brazil), 0.5% peptone (Acumedia, USA), 0.2% yeast extract (Bacto, Difco, USA), and 0.5% agar (Acumedia, USA) were equilibrated to room temperature and inoculated at the center with 10 µL of a *P. aeruginosa* suspension containing 10^8^ colony-forming units (CFU)/mL in the presence and absence (control) of subinhibitory concentrations (½ MIC) of bio-AgNPs. Plates were incubated without inversion for 24 h at 30°C (Norizan et al., 2013).

#### Swimming Motility

*P. aeruginosa* isolates were seeded on LB agar (Neogen, USA) and incubated at 37°C for 24 h. Then, one colony of each isolate was inoculated, in the presence and absence (control) of ½ MIC bio-AgNPs, on the surface of swimming agar plates containing 1.0% tryptone (Acumedia, USA), 0.5% sodium chloride (Casa Americana, Brazil), and 0.3% agar (Acumedia, USA), previously equilibrated to room temperature. Plates were incubated without inversion for 24 h at 30°C (Inoue et al., 2008).

#### Twitching Motility

*P. aeruginosa* isolates were seeded on LB agar (Neogen, USA) and incubated at 37°C for 24 h. Then, one colony of each isolate was inoculated, in the presence and absence (control) of ½ MIC bio-AgNPs, to the bottom of twitching agar plates containing 1.0% tryptone (Acumedia, USA), 0.5% yeast extract (Bacto, Difco, USA), 1.0% sodium chloride (Synth, Brazil), and 1.0% agar (Acumedia, USA). Plates were inverted and incubated at 37°C for 24 h. Subsequently, the agar was carefully removed, and the motility zone was measured to the nearest millimeter after staining with 2% crystal violet (Laborclin, Brazil) for 2 h (Otton et al., 2017). As a negative control, each isolate was inoculated in tryptone soy agar (Difco, USA) under the same conditions.

#### Biofilm Formation

Biofilm formation was analyzed on 96-well polystyrene microtiter plates by the modified crystal violet method described by Ramos-Vivas et al. (2019). *P. aeruginosa* isolates were grown on LB agar (Neogen, USA) at 37°C for 24 h. Then, 180 µL of LB broth (Neogen, USA) and 20 µL of *P. aeruginosa* suspension (initial concentration of 1.5 × 10^6^ CFU/mL) were added to each well and incubated at 37°C for 24 h in the presence and absence (control) of ½ MIC bio-AgNPs. After incubation, the supernatants were discarded, and the wells were washed three times with phosphate-buffered saline (pH 7.2), fixed with 250 µL of methanol (Merck, Germany) for 10 min, and stained with a 1.0% crystal violet aqueous solution (Merck, Germany) for 15 min. Subsequently, the crystal violet was discarded and the wells washed three times with purified water. Adhered cells were resuspended in 250 µL of 33% (v/v) glacial acetic acid (Merck, Germany). Absorbance was read spectrophotometrically (Multiskan™ FC, Thermo Fisher Scientific, USA) at 620 nm.

### Statistical Analysis

All experiments were conducted in triplicate on three separate occasions. Statistical analyses were performed using RStudio software version 1.2.5001. Graphical representations were constructed using GraphPad Prism version 8.4.2 (GraphPad Software Inc., USA).

## Results

### *P. aeruginosa* Isolates

**Figure 1:**
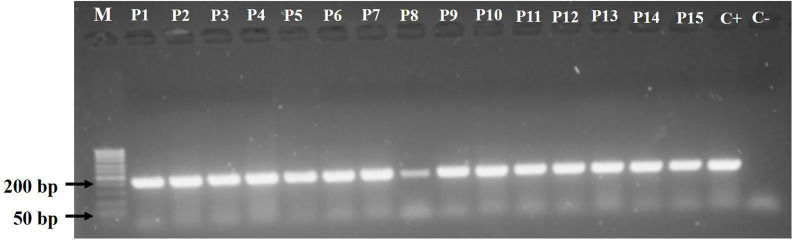
Agarose gel electrophoresis (1.0%) of *Pseudomonas aeruginosa* isolates showing amplified PCR products for the *oprL* gene (209 bp). Lane 1 (M), molecular weight marker; P1–P5, environmental isolates; P6–P10, clinical isolates obtained from non-cystic fibrosis patients; P11–P15, clinical isolates obtained from cystic fibrosis patients.

*P. aeruginosa* isolates were identified as oxidase-positive, catalase-positive, non-fermenter Gram-negative rods capable of growth on Cetrimide and M-PA-C agar. Isolates were confirmed to be *P. aeruginosa* by *oprL* gene detection (**Figure 1**).

### Characterization of Bio-AgNPs

**Figure 2:**
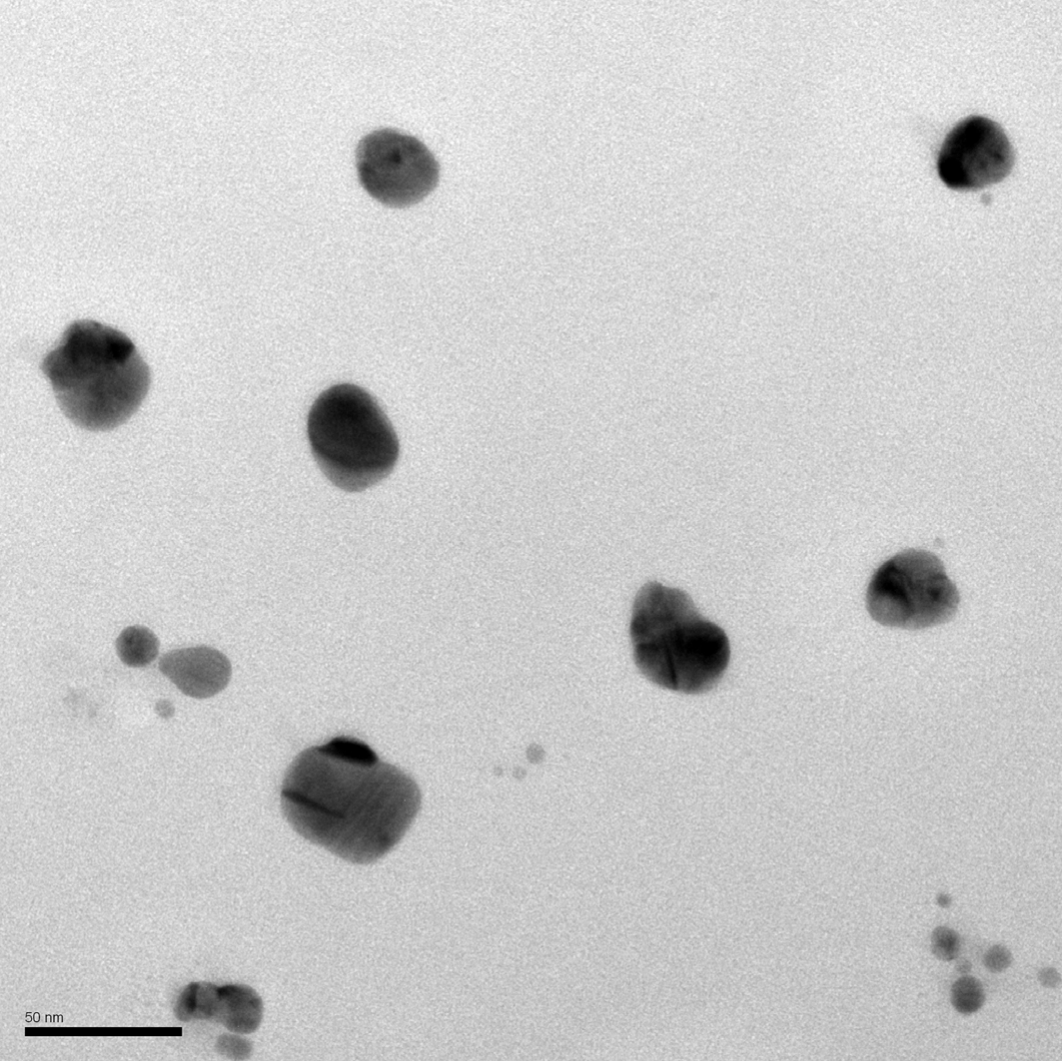
Characterization by transmission electron microscopy (TEM) of bio-AgNP synthesized by *Fusarium oxysporum* (300,000×).

The X-ray fluorescence spectra of bio-AgNPs showed a plasmonic absorption band in the visible region (near 440 nm). Nanoparticles had a spherical shape and mean size of 73.04 nm, as demonstrated by transmission electron microscopy (**Figure 2**). The surface charge (zeta potential) of bio-AgNPs was −18.77 mV.

### MIC of Bio-AgNPs

The MIC of bio-AgNPs for *P. aeruginosa* PAO1 and PA14 was 62.5 µM. MICs for environmental and clinical isolates are described in **Table 1**. Bio-AgNPs were used at ½ MIC (31.25, 15.62, or 7.81 µM) to test their action against *P. aeruginosa* motility and biofilm formation.

### Effect of Bio-AgNPs on *P. aeruginosa* Motility

We tested the sub-MIC effects of bio-AgNPs on the three types of *P. aeruginosa* motility (swarming, swimming, and twitching), measured in diameter (mm). According to **Table 2**, bio-AgNPs exerted distinct effects on the motility of reference strains PAO1 and PA14. In PAO1, bio-AgNPs significantly reduced swarming (*p* = 0.092) and swimming (*p* < 0.001). In PA14, however, nanoparticles enhanced swarming (*p* = 0.002) and twitching (*p* = 0.008) motility.

**Table 2:** Swarming, swimming, and twitching motility in reference strains (PAO1 and PA14), environmental isolates, and clinical isolates of *Pseudomonas aeruginosa* in the presence and absence of biogenic silver nanoparticles (bio-AgNPs) at ½ MIC.

Reference strainsor isolates	Type	*Swarming* (mm)		*Swimming* (mm)		*Twitching* (mm)	
		Control	bio-AgNP	Control	bio-AgNP	Control	bio-AgNP
**PAO1**	C	58.9 ± 10.4	45.7 ± 19.1**	41.0 ± 2.5	29.3 ± 3.6***	11.6 ± 0.5	11.0 ± 0.8
**PA14**	C	13.9 ± 4.8	45.0 ± 20.8**	34.7 ± 4.0	36.1 ± 3.6	13.7 ± 1.7	19.1 ± 1.1**
**P1**	E	38.8 ± 16.1	47.3 ± 22.2	43.9 ± 2.5	46.3 ± 5.2	4.2 ± 2.1	4.4 ± 1.9
**P2**	E	19.0 ± 8.2	21.4 ± 5.4	38.7 ± 6.0	48.8 ± 10.6**	5.6 ± 1.0	5.2 ± 1.3
**P3**	E	10.1 ± 0.6	15.6 ± 4.8**	41.2 ± 3.4	46.3 ± 10.6	5.0 ± 1.6	5.2 ± 1.3
**P4**	E	17.0 ± 5.2	25.6 ± 14.9	34.1 ± 7.3	34.8 ± 9.8	5.0 ± 1.1	6.3 ± 1.1**
**P5**	E	39.1 ± 20.4	44.2 ± 25.0	41.1 ± 3.6	49.7 ± 13.9	4.9 ± 0.9	5.3 ± 1.0**
**P6**	non-CF	10.9 ± 0.8	32.1 ± 29.2**	41.6 ± 7.6	43.3 ± 11.1	9.1 ± 1.9	12.1 ± 5.6*
**P7**	non-CF	8.7 ± 0.7	13.2 ± 1.2**	49.6 ± 5.0	46.2 ± 8.9	3.5 ± 0.9	5.0 ± 1.3*
**P8**	non-CF	13.3 ± 2.4	17.8 ± 4.2**	33.8 ± 3.6	44.3 ± 4.0***	5.1 ± 0.3	6.3 ± 3.3*
**P9**	non-CF	14.0 ± 1.5	28.6 ± 8.2**	41.2 ± 3.2	53.4 ± 10.6*	7.1 ± 3.3	8.1 ± 4.9
**P10**	non-CF	16.4 ± 6.4	47.8 ± 8.7***	25.2 ± 7.3	36.2 ± 6.0**	2.8 ± 1.3	3.9 ± 0.9
**P11**	CF	16.4 ± 6.9	20.6 ± 6.1	42.2 ± 2.5	48.6 ± 9.2*	7.1 ± 0.8	6.2 ± 1.7
**P12**	CF	32.7 ± 21.6	25.8 ± 13.2	42.6 ± 5.4	48.9 ± 8.1*	5.6 ± 1.1	5.3 ± 1.6
**P13**	CF	68.3 ± 15.5	73.0 ± 13.3	38.4 ± 5.1	43.0 ± 10.7	4.0 ± 0.6	3.6 ± 1.9
**P14**	CF	53.6 ± 30.0	52.9 ± 30.5	40.3 ± 8.7	39.3 ± 15.4	4.1 ± 0.7	4.8 ± 0.6**
**P15**	CF	43.9 ± 30.6	41.2 ± 27.8	45.8 ± 6.5	46.9 ± 9.1	4.1 ± 0.9	4.4 ± 1.2**

The results were obtained by the mean ± standard deviation of three independent experiments.

*P < 0.05, ** < 0.01, and *** < 0.001, comparing the control bacteria (without treatment) with the treated (bio-AgNP).

Bio-AgNP, silver nanoparticles.

C, clinical isolates.

E, environmental isolates.

Non-CF, clinical isolates obtained from non-cystic fibrosis patients.

CF, clinical isolates obtained from cystic fibrosis patients.

Most *P. aeruginosa* isolates showed an increase in swarming motility after bio-AgNP treatment, except for P12, P14, and P15 (all obtained from CF). The swarming motility diameters of six isolates (40.0%) were significantly higher (*p* < 0.050) in the presence of bio-AgNPs (**Table 2**). A similar result was observed for swimming motility (**Table 2**). This parameter increased in all isolates with bio-AgNP treatment, except P7 and P14 (non-FC and FC, respectively). Swimming motility diameters were significantly higher (*p* < 0.050) in the presence of bio-AgNPs in six isolates (40.0%). Twitching motility diameters increased (*p* < 0.050) with bio-AgNP treatment in seven isolates (46.7%).

**Figure 3:**
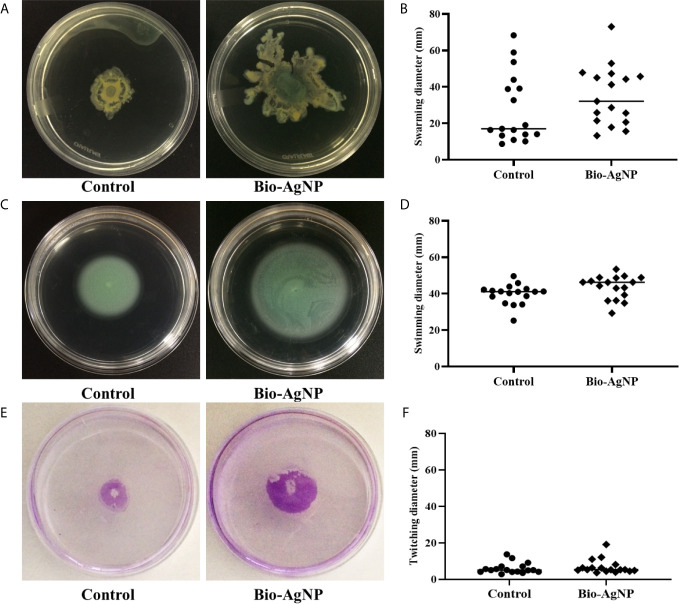
*Pseudomonas aeruginosa* motility in the absence (control) and presence of biogenic silver nanoparticles (bio-AgNPs). **(A)** Swarming motility of P2 isolate, **(B)** mean swarming motility, **(C)** swimming motility of P2 isolate, **(D)** mean swimming motility, **(E)** twitching motility of P6 isolate, and **(F)** mean twitching motility.

As shown in **Figure 3A**, the swarming motility zone of P2 in the absence of bio-AgNPs was 19.0 ± 8.2 mm, which increased to 21.4 ± 5.4 mm after bio-AgNPs were added to the culture medium. Among the tested isolates, the increase in swarming motility ranged from 6.9 to 194.5%.

The swimming motility zone of P2 was 38.7 ± 6.0 mm without bio-AgNP treatment and 48.8 ± 10.6 mm with treatment (**Figure 3C**). Bio-AgNPs increased swimming motility in *P. aeruginosa* isolates by 2.4 to 43.7%.

P6 showed a twitching motility of 9.1 ± 1.9 mm in the absence of bio-AgNPs and 12.1 ± 5.6 mm in the presence of bio-AgNPs (**Figure 3E**). Bio-AgNP treatment increased twitching motility in *P. aeruginosa* isolates by 4.0 to 42.9%. The mean swarming, swimming, and twitching diameters of *P. aeruginosa* isolates are detailed in **Figures 3B, D, F**, respectively.

### Effect of Bio-AgNPs on *P. aeruginosa* Biofilm Formation

**Figure 4:**
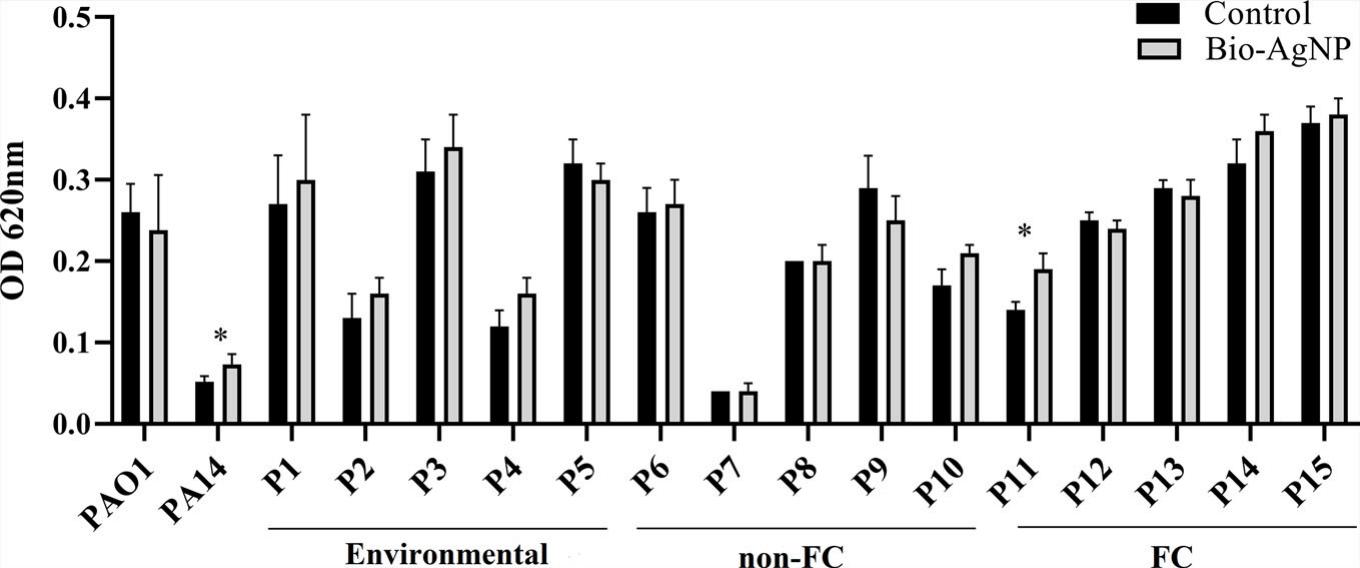
Biofilm formation capacity of reference strains, environmental isolates, and clinical isolates of *Pseudomonas aeruginosa* in the absence (control) and presence of biogenic silver nanoparticles (bio-AgNPs). Results are the mean ± standard deviation of three independent experiments. *p < 0.05 compared with the control.

Bio-AgNP treatment promoted biofilm formation in most *P. aeruginosa* isolates (73.3%), except P5, P9 (non-FC), P12, P13 (FC isolates) (**Figure 4**). Similar effects were exerted on PAO1 and PA14. However, a significant increase in biofilm formation was only observed in the PA14 reference strain (*p* = 0.040) and the P11 isolate (*p* = 0.034), obtained from a patient with cystic fibrosis (**Figure 4**).

## Discussion

The antibacterial activity of bio-AgNPs against *Klebsiella pneumoniae*, *Staphylococcus aureus*, *Escherichia coli*, *Acinetobacter baumannii*, and *Salmonella* Enteritidis at inhibitory concentrations was previously described by Scandorieiro et al. (2016) and Figueiredo et al. (2019). Few studies, however, have investigated the subinhibitory effects of AgNPs on clinical isolates or environmental microorganisms (Yang and Alvarez, 2015; Garuglieri et al., 2016; Grün et al., 2018).

Motility is fundamental for bacterial colonization of certain surfaces; it allows microorganisms to move toward more favorable environments and away from unfavorable conditions. Motility is also important for pathogenicity, as it is involved in host cell adhesion, colonization, biofilm formation, and survivability (Sun et al., 2018).

In the present study, the PAO1 reference strain showed a significant reduction in swarming and swimming motility with bio-AgNP treatment. In contrast, PA14 showed a significant increase in swarming and twitching motility. These reference strains differ in origin and genomic characteristics. *P. aeruginosa* PAO1, isolated from a wound, is a moderately virulent strain belonging to a relatively rare clonal group. PA14 is highly virulent in several infection models and is part of the most common clonal group worldwide. Among other factors, differences between strains may be due to the presence of two pathogenicity islands (PAPI-1 and PAPI-2) in PA14 but not in PAO1 (Harrison et al., 2010; Mikkelsen et al., 2011). PAPI-1 encodes several virulence-associated factors, including type IVb pili (*pilS2* gene) (Bahramian et al., 2019) and PvrR, a response regulator involved in the synthesis of bacterial biofilms (Guła et al., 2019). PAPI-2 encodes ExoU cytotoxin, a potent phospholipase (Harrison et al., 2010). Researchers have shown that exclusion of one or both pathogenicity islands affects the virulence of PA14 in murine models of acute pneumonia and bacteremia (Harrison et al., 2010). According to Mikkelsen et al. (2011), even though PA14 has pathogenicity islands that are not found in PAO1, the presence or absence of specific gene clusters is not predictive of virulence. The referred study found that PA14 has an acquired mutation in the *ladS* gene that has a deleterious impact on biofilm formation but promotes the activity of type III secretion systems and cytotoxicity in mammalian cells. This could explain why PA14 had a lower biofilm-forming capacity than PAO1 in the present study.

Our results showed that about 40% of isolates increased swarming, swimming, and twitching activity after treatment with bio-AgNPs at sub-MIC. Villa et al. (2012) demonstrated that sublethal concentrations of oxidizing biocides increase swarming and swimming motility in the soil bacterium *Azotobacter vinelandii*, representing a strategy to mitigate adverse conditions. Ortega-Calvo et al. (2011) also presented evidence that AgNPs induce a repellent response in *Pseudomonas putida*. The mechanism that causes repellence remains unknown but it is believed to involve physiological changes caused by accumulation of AgNPs in the cell membrane and/or within cells, modifying microbial performance. The size of AgNPs also seems to influence repellence. Another study revealed that subinhibitory concentrations of AgNPs stimulated swarming and swimming in *E. coli* and *Bacillus subtilis* under aerobic and anaerobic conditions (Garuglieri et al., 2016).

Singh et al. (2019) investigated the effects of AgNPs and silver ions on *P. aeruginosa* PAO1. Treatment with 3.12 µg/mL AgNP increased the expression of motility-related genes, such as *bswR* (swarming motility regulator) and *pilG* (twitching motility protein), and the activation of flagellum-related genes (*flgC*, *fliG*, *fliH*, and *fliP*).

In this study, after 24 h of incubation with ½ MIC bio-AgNPs, there was an increase in biofilm production in the reference strain PA14 and isolate P11. Such an increase can be explained by one of the mechanisms of action of AgNPs in bacterial cells: formation of reactive oxygen species (ROS), which cause oxidative stress (Wang et al., 2017). The high ROS concentrations occurring under oxidative stress may lead to adverse modifications in cellular components and damage to proteins, DNA, and lipids (Su et al., 2019). Villa et al. (2012) reported that oxidative stress in *A. vinelandii* increased with oxidizing biocide treatment. It is probable that high oxidative stress exerted selective pressure at the initial stage of biofilm development, enhancing biofilm formation capacity.

*Pseudomonas* biofilms are composed of at least three types of polysaccharides: Psl, Pel, and alginate. Research has shown that *pslA* expression decreased in the presence of AgNPs and that expression of *pslD* (a gene encoding an outer membrane protein) decreased in the presence of silver ions. On the other hand, AgNPs and silver ions increased the expression of genes associated with glycosyltransferases, *pslC*, *pslL*, and *pslE* (Wzz/Wzc-like protein) (Singh et al., 2019).

Guo et al. (2019) observed that subinhibitory doses of AgNPs enhanced polysaccharide and protein production in *P. aeruginosa* CCM 3955 compared with the control, significantly altering biofilm structure. The major form of protection of bacteria against low AgNP concentrations was to increase exopolysaccharide secretion.

According to Yang and Alvarez (2015), exposure of *P. aeruginosa* PAO1 to sublethal AgNP concentrations improved biofilm development and increased expression of genes related to QS (*lasI* and *lasR*). *P. aeruginosa* has three known QS systems: las, rhl, and PQS (Lee and Zhang, 2015; Cornelis, 2020). QS systems allow communication between microorganisms through the production and diffusion of chemical or signaling molecules that control a variety of physiological functions, such as biofilm formation and motility activity (Saeki et al., 2020). Rasamiravaka et al. (2015) reported that the three QS systems of *P. aeruginosa* play important roles in biofilm formation and development.

Contrasting results were obtained by Hussain et al. (2019) when testing the effects of AgNPs (10–50 nm) on different microorganisms, including *P. aeruginosa* PAO1. At subinhibitory concentrations (1/16, 1/8, 1/4, and 1/2 MIC), there was a 42–81% reduction in swarming motility and a 22–79% reduction in biofilm formation. Another study showed that subinhibitory concentrations of gold nanoparticles (mean size of 53 nm) significantly reduced (*p* < 0.01) swarming, swimming, twitching, and biofilm formation (Khan et al., 2019). These differences may be related to nanoparticle size. Liao et al. (2019) showed that larger nanoparticles have lower antibacterial activity than smaller nanoparticles. The bio-AgNPs prepared in this study had a mean size of 75 nm. Other studies have shown that size may not be a determinant of virulence factor regulation. Garuglieri et al. (2016) showed that treatment with AgNPs sized 14 ± 0.3 nm increased motility and Grün et al. (2018) found that treatment with 30–70 nm AgNPs enhanced biofilm formation.

Currently, there are several commercial AgNP products widely used in the medical and food industries and water treatment plants (McNamara and Tofail, 2017; Gadkari et al., 2018; Jaiswal et al., 2019). This wide use implies that metal compounds are constantly released into the aquatic environment through sewage and wastewater disposal (Das et al., 2012; Singh et al., 2019). The release of AgNPs, even at subinhibitory concentrations, is a cause of environmental concern (Yang and Alvarez, 2015; Grün et al., 2018).

Overall, it can be inferred that the increase in phenotypic expression of biofilm and bacterial motility after exposure to bio-AgNPs may be associated with chemical signaling, as these virulence factors are regulated by the QS system. Therefore, further research is needed on the action of AgNPs in the QS system of *P. aeruginosa* for a better understanding of their effects at subinhibitory concentrations. Moreover, it is necessary to explore other forms of AgNP use with the aim of reducing antimicrobial resistance as well as environmental impacts.

Upon exposure to subinhibitory concentrations of bio-AgNPs, the PA14 reference strain and some *P. aeruginosa* isolates showed increased swarming, swimming, and twitching motility and biofilm formation. Unfortunately, some studies have also described that low doses of nanoparticles can significantly induce biofilm formation and virulence factors *in vitro*. This increase in bacterial exposure to nanoparticles is a cause for concern, given all the adverse effects that have not been fully clarified. Therefore, the use of subinhibitory concentrations bio-AgNPs especially in therapeutic applications must be performed with precaution.

These results are an initial contribution to broaden our understanding of the effects of subinhibitory concentrations of bio-AgNPs on bacterial behavior, an important but little-studied phenomenon. However, it is still necessary to elucidate the regulation of virulence factors in bio-AgNP-treated bacteria. Further research on the interference mechanisms of subinhibitory concentrations of bio-AgNPs in *P. aeruginosa* QS systems is recommended.

## Data Availability Statement

The raw data supporting the conclusions of this article will be made available by the authors, without undue reservation.

## Author Contributions

EKS designed the study, interpreted the clinical data, and wrote the paper. AYY and LAA analyzed the virulence factors of *Pseudomonas aeruginosa.* LA performed the genomics experiments. DOG and RLBS discussed the results and implications and wrote the manuscript. RKTK and GN interpreted the clinical data. All authors contributed to the article and approved the submitted version.

## Conflict of Interest

The authors declare that the research was conducted in the absence of any commercial or financial relationships that could be construed as a potential conflict of interest.
